# Xanthine Oxidase Inhibitor Allopurinol Prevents Oxidative Stress‐Mediated Atrial Remodeling in Alloxan‐Induced Diabetes Mellitus Rabbits

**DOI:** 10.1161/JAHA.118.008807

**Published:** 2018-05-02

**Authors:** Yajuan Yang, Jianping Zhao, Jiuchun Qiu, Jian Li, Xue Liang, Zhiwei Zhang, Xiaowei Zhang, Huaying Fu, Panagiotis Korantzopoulos, Konstantinos P. Letsas, Gary Tse, Guangping Li, Tong Liu

**Affiliations:** ^1^ Tianjin Key Laboratory of Ionic‐Molecular Function of Cardiovascular Disease Department of Cardiology Tianjin Institute of Cardiology Second Hospital of Tianjin Medical University Tianjin China; ^2^ First Department of Cardiology University of Ioannina Medical School Ioannina Greece; ^3^ Laboratory of Cardiac Electrophysiology Second Department of Cardiology Evangelismos General Hospital of Athens Greece; ^4^ Department of Medicine and Therapeutics Faculty of Medicine Chinese University of Hong Kong China; ^5^ Li Ka Shing Institute of Health Sciences Faculty of Medicine Chinese University of Hong Kong China

**Keywords:** allopurinol, atrial fibrillation, calcium signaling, Diabetes mellitus, mitochondria, oxidative stress, xanthine oxidase, Oxidant Stress, Calcium Cycling/Excitation-Contraction Coupling, Animal Models of Human Disease, Fibrosis

## Abstract

**Background:**

There are several mechanisms, including inflammation, oxidative stress and abnormal calcium homeostasis, involved in the pathogenesis of atrial fibrillation. In diabetes mellitus (DM), increased oxidative stress may be attributable to higher xanthine oxidase activity. In this study, we examined the relationship between oxidative stress and atrial electrical and structural remodeling, and calcium handling abnormalities, and the potential beneficial effects of the xanthine oxidase inhibitor allopurinol upon these pathological changes.

**Methods and Results:**

Ninety rabbits were randomly and equally divided into 3 groups: control, DM, and allopurinol‐treated DM group. Echocardiographic and hemodynamic assessments were performed in vivo. Serum and tissue markers of oxidative stress and atrial fibrosis, including the protein expression were examined. Atrial interstitial fibrosis was evaluated by Masson trichrome staining. I_CaL_ was measured from isolated left atrial cardiomyocytes using voltage‐clamp techniques. Confocal microscopy was used to detect intracellular calcium transients. The Ca^2+^ handling protein expression was analyzed by Western blotting. Mitochondrial‐related proteins were analyzed as markers of mitochondrial function. Compared with the control group, rabbits with DM showed left ventricular hypertrophy, increased atrial interstitial fibrosis, oxidative stress and fibrosis markers, I_CaL_ and intracellular calcium transient, and atrial fibrillation inducibility. These abnormalities were alleviated by allopurinol treatment.

**Conclusions:**

Allopurinol, via its antioxidant effects, reduces atrial mechanical, structural, ion channel remodeling and mitochondrial synthesis abnormalities induced by DM‐related increases in oxidative stress.

**Clinical Perspective**

**What Is New?**

- Allopurinol, a xanthine oxidase inhibitor, reduces atrial mechanical, structural, ion‐channel remodeling and mitochondrial synthesis abnormalities caused by diabetes mellitus‐related oxidative stress.

**What Are the Clinical Implications?**

- Xanthine oxidase activation is related to diabetes mellitus‐induced atrial fibrillation. Allopurinol may act as a potential upstream therapy for atrial fibrillation in the setting of diabetes.

Atrial fibrillation (AF) is one of the most common arrhythmias observed in medical practice and is responsible for significant cardiovascular morbidity and mortality worldwide.^1^, ^2^ Although the pathophysiological mechanisms linking diabetes mellitus (DM) to AF development have not been completely elucidated. Autonomic, electrical and structural remodeling involving activation of proinflammatory pathways, abnormalities of calcium (Ca^2+^) homeostasis, as well as oxidative stress‐mediated damage play important roles.^3^, ^4^, ^5^, ^6^

Xanthine oxidase (XO), a key enzyme of purine catabolism, is responsible for the generation of reactive oxygen species (ROS) via several pathways that include nitric oxide (NO) and calcium signaling.^7^, ^8^ Intracellular Ca^2+^ handling is a critical regulator of action potential duration, and mechanical activity of cardiomyocytes via excitation‐contraction coupling.^9^ Abnormalities in its homeostasis can therefore reduce cardiac output and potentially leading to mortality but also initiate arrhythmogenic triggers in the atrial or ventricular myocardium.^10^, ^11^, ^12^ Previous studies have reported that XO inhibition has beneficial effects on cardiac remodeling,^13^, ^14^, ^15^ mechano‐energetics^16^ and endothelial function^17^ both in experimental and clinical studies. However, the role of XO inhibition on atrial remodeling in AF remains unclear. Therefore, our study investigated the potential beneficial effects of allopurinol, a XO inhibitor, on atrial remodeling in alloxan‐induced diabetic rabbits.

## Materials and Methods

### Data Availability

The authors confirm that the data, analytic methods, and study materials will be made available to other researchers for purposes of reproducing the results or replicating the procedure. Material will be available from Tianjin Institute of Cardiology, Second Hospital of Tianjin Medical University, which is responsible for maintaining availability upon request to the corresponding author.

This study was approved by the Experimental Animal Administration Committee of Tianjin Medical University and Tianjin Municipal Commission for Experimental Animal Control and is fully compliant with National Institutes of Health guidelines from the United States.

### Experimental Animals

A total of 90 male and female Japanese rabbits (weighing 1.7–2.5 kg at the beginning of the study) were obtained from Beijing Medical Animals Research Institute (Beijing, China). They were randomly and equally divided into the control group (C, n=30), alloxan‐induced group with DM (DM, n=30) and allopurinol‐treated group with DM (ALLO, n=30). Ten rabbits from each group were used for echocardiographic examination, hemodynamic‐, histological‐ and electrophysiological studies, oxidative stress marker measurements, Western blot analyses, I_CaL_ recordings, and intracellular Ca^2+^ imaging. For the induction of diabetes mellitus, alloxan monohydrate (Sigma, Saint Louis, MO, USA) was dissolved to sterile normal saline to achieve a concentration of 5% (w/v), and a dose of 120 mg/kg was immediately administered intravenously via the marginal ear vein. The presence of diabetes mellitus was confirmed 48 hours later by blood glucose levels ≥14 mmol/L (once) or ≥11 mmol/L (twice). Subsequently, blood glucose level monitoring was performed weekly using the glucometer Optium Xceed (Abbott, Bedford, MA, USA). Rabbits in the ALLO group received allopurinol (60 mg/kg per day) orally for 8 weeks.

### Echocardiographic Examination

After 8 weeks, rabbits were anesthetized with sodium pentobarbital (3%; 30 mg/kg), and then were placed on the table on the left lateral decubitus position. Echocardiographic parameters were obtained in the parasternal long‐axis view using a GE Vingmed machine (Vivid 7/Vingmed General Electric) equipped with a 7.5‐MHz standard pediatric probe. Both two‐dimensional and M‐mode echocardiography were performed in the parasternal long‐axis view. The measurements included interventricular septal thickness and left ventricular posterior wall thickness, left ventricular cavity size end‐diastolic dimensions, left ventricular cavity size end‐systolic dimensions, and left atrial anteroposterior diameter. Measurements of LV ejection fraction were recorded according to international standards. The mean of 3 measurements were used for subsequent analysis.

### Electrophysiological Studies

After echocardiography, median sternotomy was performed, following which the heart was quickly removed and placed in cold perfusion fluid (4°C). The aorta was cannulated and connected to a Langendorff perfusion system filled with warmed (37°C±0.5°C) Tyrode's solution. The perfusion pressure was maintained at 65 to 75 mm Hg, resulting in an initial coronary flow of 50 to 60 mL/min. Tyrode's solution consisted of the following (mmol/L): NaCl 130, KCl 5.6, NaHCO_3_ 24.2, CaCl_2_ 2.2, MgCl_2_ 0.6, NaH_2_PO_4_ 1.2, and glucose 12. The solution reservoir was saturated with 95% O_2_ and 5% CO_2_ and the pH of was set at 7.4.

Four sets of silver bipolar electrodes were placed on the epicardial surface of high right atrium, high left atrium, left atrial appendage, and right ventricular apex. The S2 extra‐stimulus was delivered after a drive‐train of 8 S1S1 stimuli. The S1S2 interval was decreased by an interval of 2 ms until atrial refractoriness was reached. The atrial effective refractory period (AERP), which was defined as the longest S1S2 interval that failed to elicit atrial activity. AERP dispersion (AERPD) was defined as the difference between the longest AERP and the shortest AERP from 4 different sites. The Wenckebach cycle length of atrial–ventricular conduction was measured by right atrial incremental pacing. The interatrial conduction time, the duration from the high right atrium pacing stimulus to the beginning of high left atrium stimulus, was measured during high right atrium (at a pacing cycle length of 250 ms) pacing. AF vulnerability was tested by burst pacing (cycle length of 50 ms) for 1 second, 5 times at 30 seconds interval at amplitude of 5 V. AF was defined as rapid, irregular atrial response longer than 1000 ms. All epicardial ECGs were amplified with a custom‐made amplifier and recorded with a custom‐made computer software program (Electrophysiological Recording System, TOP‐2001, HTONG Company, Shanghai, China).

### Hemodynamic Studies

After 8 weeks, rabbits were anesthetized with 3% sodium pentobarbital (30 mg/kg) (n=10 for each group). Bipolar electrodes were placed on four limbs to obtain ECG tracings. The neck was cut in the middle and right carotid artery was isolated with great care. Following blood collection from the carotid artery for serum biochemical tests, a cannula was inserted into the right carotid artery to monitor aortic systolic and diastolic blood pressure (SBP and DBP), which were recorded after a stabilization period of 5 to 10 minutes for 3 times by 20‐second interval. Subsequently, the cannula was moved slowly through the aortic valve to left ventricle to measure left ventricle end diastolic pressure (LVEDP). The ECG and BP were continuously monitored using a custom‐made computer software program (Electrophysiological Recording System, TOP‐2001, HTONG Company).

### Histological Studies

After hemodynamic studies, the hearts were rapidly excised. The wet weight (mg) of the entire heart, left atrium, and left ventricle were measured after washing with cold phosphate–buffered saline (PBS). These were expressed relative to the total body weight (g). Isolated LA tissues were divided into 2 parts, for histological study and for Western blotting. The left atrium (LA) tissue was placed in 4% paraformaldehyde and embedded in paraffin. Tissue samples were sliced at 5‐μm thickness and stained with Masson's trichrome. The microscopic images were scanned into a personal computer and quantitatively analyzed with Photoshop 7.0 (Adobe, San Jose, CA, USA). Interstitial fibrosis was quantified on the basis of a color discrimination algorithm and expressed as a percentage of the reference tissue area (the left atrial interstitial collagen volume fraction). Blood vessels were excluded from quantification. The mean value of the 3 fields in each section was used for the analysis. Histological examination was performed by the investigators who were blinded to treatment assignment.

### Serum Biochemical and Oxidative Stress Parameters

Blood samples were collected from the carotid artery of rabbits at the end of the experiment and then centrifuged at 1308 g for 10 minutes. Plasma was separated for later tested. Blood urea nitrogen, creatinine, cholesterol, triglyceride, low‐density lipoprotein cholesterin, high‐density lipoprotein cholesterol, and fasting insulin levels were measured using automated techniques. Serum levels of oxidative stress markers, including nitric oxide (NO), malondialdehyde (MDA) and superoxide dismutase, were assessed by commercially available kits (Nanjing Jiancheng Bioengineering Institute, Jiangsu Province, China) according to the manufacturer's instructions. Xanthine oxidase (XO) activity in serum was determined using a xanthine oxidase activity quantitative colorimetric/fluorimetric assay kit (Nanjing Jiancheng Bioengineering Institute, Jiangsu Province, China). The principle for measurement of XO activity is that hypoxanthine can transform into xanthine under the catalysis of XO, during which superoxide anion free radicals were generated. The free radicals were combined with electron acceptor and developer to form conjugate, based on which XO activity can be calculated.

### Western Blot Analyses

LA tissue samples of all groups were pulverized in liquid nitrogen and extracted by 1 mL RIPA lysis buffer. The lysates were centrifuged at 12 000*g* for 15 minutes before the 5× SDS‐PAGE sample loading buffer was added to each lysate, which was subsequently boiled for 3 minutes and then electrophoresed in SDS‐polyacrylamide gels. After transferred into the Polyvinylidene fluoride (PVDF) sheets (Millipore, USA), the proteins were separately incubated with primary antibodies, followed by incubation with appropriate peroxidase‐conjugated secondary antibodies. Equal protein loading of the samples was verified by β‐actin. The reactions were visualized with Western LightningTM Chemiluminescence Reagent (Millipore). The blots were exposed to autoradiographic film (Fujifilm Holdings Corp., Japan) according to the manufacturer's instructions. Results were approved by repeating the reactions 3 times. Fibrosis‐related factors included transforming growth factor‐β1 (TGF‐β1), p38 mitogen‐activated protein kinases (p38), phosphorylated p38 mitogen‐activated protein kinases (P‐p38), c‐Jun N‐terminal kinase (JNK), phosphorylated c‐Jun N‐termial kinase (P‐JNK), extracellular signal‐regulated kinase (ERK), phosphorylated extracellular signal‐regulated kinase (P‐ERK) as well as nuclear factor kappa‐B (NF‐κB) were measured. Xanthine oxidase (XO) and manganese superoxide dismutase (MnSOD) were measured to evaluate oxidative stress in cardiomyocytes. We analyzed alpha 1C subunit of L‐type calcium channel (Cav1.2), ryanodine Receptor 2 (RyR2), sarcoplasmic reticulum Ca^2+^‐ATPase2a (SERCA2a), Phospholamban (PLB), phosphorylated form of phospholamban (P‐PLB) as well as FK506‐binding protein 12.6 (FKBP12.6) for purpose of observing the influence of diabetes mellitus on remodeling of proteins involved in calcium homeostasis. Furthermore, we analyzed nuclear respiratory factor‐1, mitochondrial transcription factor A, dynamin‐related protein 1 (Drp‐1) as well as mitofusin 1 (mfn1) to observe the impact of diabetes mellitus on mitochondrial function. Detailed information of primary antibodies are detailed in Table S1.

### Atrial Myocyte Isolation

Atrial cardiomyocytes were isolated enzymatically as previously described.^18^ After anesthetized with sodium pentobarbital (3%; 30 mg/kg), the heart was quickly removed from the torso and was placed in cold perfusion fluid (4°C). The heart was then mounted onto Langendorff‐perfusion apparatus filled with warmed (37±0.5°C) Ca^2+^‐free Tyrode's solution at 30 mL/min for 15 minutes, followed by a perfusion containing collagenase (0.075%, CLS II, Worthington Biochemical, Lakewood, CO, USA) and 0.2% bovine serum albumin (Sigma Chemical Co., St. Louis, MO, USA) for another 20 minutes. The atria were cut into small pieces and maintained at a 37°C high‐K^+^ storage solution (KB solution). Atrial cardiomyocytes were filtered on gauze and allowed to sediment by gravity for 5 minutes, followed by superfusion with Tyrode's solution (3 mL/min) for 5 minutes, after which cells were suspended in Tyrode's solution with 1 mmol/L CaCl_2_. Freshly isolated atrial cardiomyocytes were plated in culture dishes and stored at room temperature until use.

### I_CaL_ Recording

The whole‐cell configuration of the patch‐clamp technique was used to record I_CaL_ at room temperature (24–26°C). The patch pipette resistance was 1.0 to 2.0 MΩ when filled with pipette solution (composition in mmol/L: CsCl 125, MgATP 5, EGTA 15, TEA‐Cl 20 HEPES/Cs+ 10, adjusted with CsOH to pH 7.2). I_CaL_ and action potentials (APs) were recorded and filtered at 0.5 kHz using a low pass filter. The sampling frequency was set at 0.2 Hz for I_CaL_. After seal formation and membrane rupture, a single 10 mV hyperpolarizing pulse (from −50 mV) in the extracellular solution over a voltage range of −40 to +60 mV was applied to determine the current‐voltage relation for I_CaL_.

### Intracellular Ca^2+^ Imaging

[Ca^2+^]_i_ transients were recorded in intact myocytes previously loaded with the fluorescent Ca^2+^ dye Fluo‐4AM (15 μmol/L, DOJINDO, Japan) for 15 minutes and then rinsed twice in Tyrode's solution to wash away residual dye. Cardiomyocytes were electrically stimulated at 1 and 2 Hz by field stimulation using two parallel platinum electrodes. Confocal microscopy (Olympus FV1000, ×20 oil immersion objective with a 0.75NA) was used to obtain the images of cardiomyocytes exhibiting Ca^2+^ by scanning the cell with anargon laser. Fluo‐4AM was excited at 488 nm, and emitted fluorescence was collected at >505 nm. The fluorescence values (F) were normalized by the basal fluorescence (F0) to obtain the fluorescence ratio (F/F0). Data analysis was performed with GraphPad Prism (Version 6.0).

### Statistical Analysis

Statistical analysis was performed using SPSS 22.0 statistical software. Data are presented as mean±SD. Comparisons between the groups were analyzed for statistical significance using the chi‐squared test and ANOVA followed by Dunnett's test. *P*<0.05 were considered statistically significant.

## Results

### Baseline and Echocardiographic Characteristics

The baseline characteristics of the included animals and echocardiographic parameters are shown in Table 1. Compared with controls, rabbits with diabetes showed higher glucose levels (5.55±0.27 mmol/L versus 16.15±8.27 mmol/L, *P*<0.05) that were partially reduced by allopurinol treatment (16.15±8.27 mmol/L versus 11.63±5.84 mmol/L, *P*>0.05). There was no significant difference in systolic blood pressure (86.71±14.34, 96.43±11.27, and 110.71±29.05, respectively, *P*>0.05) or diastolic blood pressure (69.86±15.93, 65.14±31.77, and 91.57±36.66 mm Hg, *P*>0.05) observed between control, DM, and allopurinol groups. Atrium weight ratio (0.26±0.08 versus 0.31±0.02, *P*<0.05), LV weight ratio (1.51±0.93 versus 1.74±0.15, *P*<0.05) and heart weight ratio (2.18±0.69 versus 2.39±0.18, *P*<0.05) were increased in the DM group compared with the control group, whilst allopurinol restored these changes partially (0.31±0.02 versus 0.29±0.03, 1.74±0.15 versus 1.61±0.13, 2.39±0.18 versus 2.24±0.25, *P*>0.05).

**Table 1:** Baseline Characteristics and Echocardiographic Parameters in Experimental Groups

	Control Group (n=10)	DM Group (n=10)	ALLO Group (n=10)
Weight, kg	2.56±0.21	2.59±0.28	2.79±0.30
Glucose level at 8 wks, mmol/L	5.55±0.27	16.15±8.27^a^	11.63±5.84^a^
HR, bpm	176.40±15.46	168.40±10.83	164.40±15.13
SBP, mm Hg	86.71±14.34	96.43±11.27	110.71±29.05
DBP, mm Hg	69.86±15.93	65.14±31.77	91.57±36.66
LVEDP, mm Hg	8.04±3.33	2.84±2.54	3.88±3.24
LAD, mm	7.26±0.57	8.57±0.95	7.85±1.27
Septal wall thickness, mm	1.71±0.13	2.08±0.23^a^	1.91±0.19
Posterior wall thickness, mm	1.65±0.15	2.07±0.35^a^	1.90±0.20
LV end diastolic diameter, mm	12.42±0.84	14.38±1.00^a^	13.55±1.30
LV end systolic diameter, mm	6.82±0.52	8.61±1.47^a^	8.06±1.09
LV ejection fraction, %	70.23±7.81	76.25±11.54	69.57±11.88
E, cm/s	55.62±9.03	66.68±7.94	50.08±14.95^b^
A, cm/s	41.17±13.67	38.40±9.12	39.18±12.93
E/A	1.38±0.27	1.85±0.68	1.37±0.53
Heart weight ratio, 1/1000	2.18±0.69	2.39±0.18^a^	2.24±0.25
Atrium weight ratio, 1/1000	0.26±0.08	0.31±0.02^a^	0.29±0.03
LV weight ratio, 1/1000	1.51±0.93	1.74±0.15^a^	1.61±0.13

Values are mean±SD. ALLO indicates allopurinol; DBP, aortic diastolic blood pressure; HR, heart rate; IVST, interventricular septal wall thickness; LAD, left atrial anteroposterior diameter; LV, left ventricular; LVEDP, left ventricular end‐diastolic pressure; LVEF,left ventricular ejection fraction; SBP, aortic systolic blood pressure.

a Compared with the control group *P*<0.05.

b Compared with the DM group *P*<0.05.

**Figure 1:**
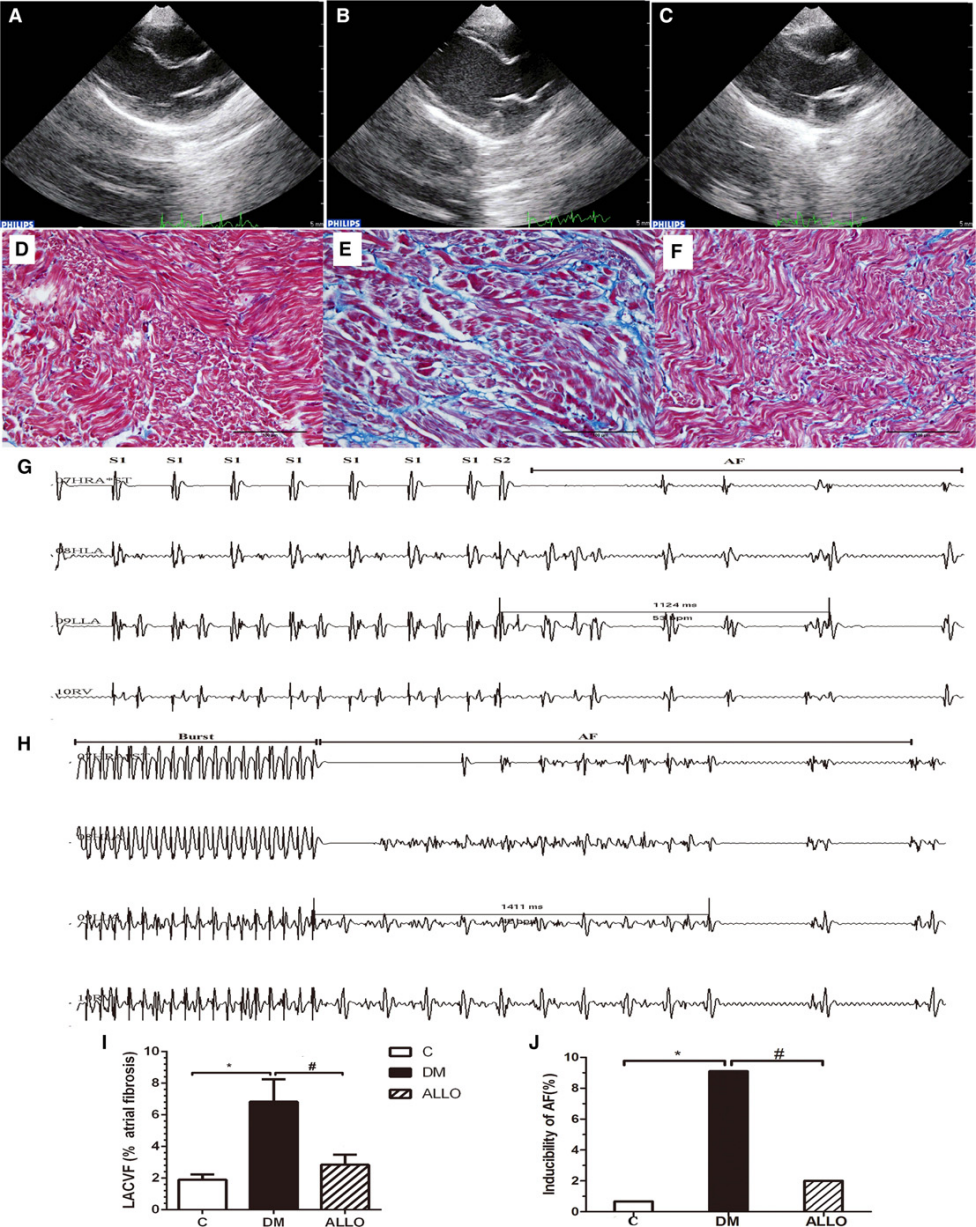
Representative echocardiographic images of the atria (A through C), left atrial interstitial fibrosis (D through F, I) and AF inducibility (G, H, and J) for the 3 groups: Control group (A and D); DM group (B and E); ALLO group (C and F). AF induced by S1S2 pacing (G) and burst pacing (H). I, The percentage of left atrial interstitial fibrosis in each group. J, The inducibility of AF in the 3 groups. * and ^#^ indicate significant difference at *P*<0.05 when compared with the control group and DM group, respectively. AF indicates atrial fibrillation; ALLO, allopurinol; DM, diabetes mellitus; LACVF, left atrial interstitial collagen volume fraction; NS, not significant.

Representative images from echocardiographic studies are shown in Figures 1A through 1C. Interventricular septal wall thickness (1.71±0.13 mm versus 2.08±0.23 mm, *P*<0.05), left ventricular posterior wall thickness (1.65±0.15 mm versus 2.07±0.35 mm, *P*<0.05), left ventricular cavity size end‐diastolic dimensions (12.42±0.84 mm versus 14.38±1.00 mm, *P*<0.05) and left ventricular cavity size end‐systolic dimensions (6.82±0.52 mm versus 8.61±1.47 mm, *P*<0.05) were significantly increased in the DM group compared with the control group, which were partially prevented by allopurinol treatment (interventricular septal thickness, 2.08±0.23 mm versus 1.91±0.19 mm; left ventricular posterior wall thickness, 2.07±0.35 mm versus 1.90±0.20 mm; left ventricular cavity size end‐diastolic dimensions, 14.38±1.00 mm versus 13.55±1.30 mm; left ventricular cavity size end‐systolic dimensions, 8.61±1.47 mm versus 8.06±1.09 mm, *P*>0.05). By contrast, no difference in LA diameter (7.26±0.57, 8.57±0.95, and 7.85±1.27 mm, respectively, *P*>0.05) and LV ejection fraction (70.23±7.81%, 76.25±11.54% and 69.57±11.88%, respectively, *P*>0.05) was observed between the 3 groups.

### Left Atrial Interstitial Fibrosis

The findings from Masson's trichrome staining are shown in Figures 1D through 1F. LA interstitial fibrosis and left atrium collagen volume fraction were increased in the DM group compared with the control group (1.90% versus 6.83%, *P*<0.05), which was attenuated by allopurinol (Figure 1I, 6.83% versus 2.85%, *P*<0.05).

### Electrophysiological Studies

The findings from electrophysiological studies are shown in Table 2. Interatrial conduction time ( 22.10±3.67 ms versus 27.30±2.83 ms, *P*<0.05) and atrial effective refractory period dispersion (AERPD, 21.00±4.35 ms versus 26.80±5.67 ms, *P*<0.05) were significantly prolonged and atrioventricular Wenckebach cycle length (cycle length of atrial–ventricular conduction, 159.40±16.44 ms versus 138.90±18.27 ms, *P*<0.05) were significantly shorten in diabetic rabbits, which were attenuated by the treatment with allopurinol (interatrial conduction time, 27.30±2.83 ms versus 23.90±2.6 ms, AERPD, 26.80±5.67 ms versus 22.80±5.98 ms, cycle length of atrial–ventricular conduction, 138.90±18.27 ms versus 153.40±14.34 ms, *P*<0.05). By contrast, no significant differences in AERP values measured from the high left atrium, low left atrium, high right atrium were found between these groups (*P*>0.05). Results from S1S2 and burst pacing are shown Figures 1G and 1H, respectively, demonstrating episodes of induced AF. The incidence of induced AF is significantly higher in the DM compared with the control group (41/450 versus 3/450 or 9.11% versus 0.67%. The denominator of 450 indicates 450 the total number of pacing during observation), and significantly lowered by allopurinol treatment (41/450 versus 9/450 or 9.11% versus 2.00%) (Figure 1J).

**Table 2:** Hemodynamic and Electrophysiological Studies

	Control Group (n=10)	DM Group (n=10)	ALLO Group (n=10)
IACT, ms	22.10±3.67	27.30±2.83^a^	23.90±2.6^b^
AVWCL, ms	159.40±16.44	138.90±18.27^a^	153.40±14.34^b^
HRAERP, 250 ms	86.20±12.16	90.00±14.36	84.00±8.60
HRAERP, 200 ms	84.80±7.84	96.00±16.47	86.60±7.49
HLAERP, 250 ms	84.40±7.65	93.40±17.33	88.40±11.27
HLAERP, 200 ms	81.80±7.74	93.80±12.27	89.40±11.08
LLAERP, 250 ms	87.20±9.30	97.80±13.61	90.80±9.94
LLAERP, 200 ms	89.40±7.43	94.20±14.74	87.60±11.99
AERPD, ms	21.00±4.35	26.80±5.67^a^	22.80±5.98^b^

Values are mean±SD. AERPD indicates atrial effective refractory periods dispersion; AVWCL, Wenckebach cycle length of A‐V conduction; DM, diabetes mellitus; HLAERP, high left atrium effective refractory period; HRAERP, high right atrium effective refractory period; IACT, interatrial conduction time; LLAERP, low left atrium effective refractory period.

a Compared with the control group *P*<0.05.

b Compared with the DM group *P*<0.05.

### Serum Oxidative Stress Markers, Biochemical Parameters, and Western Blot Analyses

Serum biochemical parameters and oxidative stress marker levels are shown in Table 3. No significant differences in blood urea nitrogen, creatinine, TG, TC, high‐density lipoprotein cholesterol, and low‐density lipoprotein cholesterol were observed among the 3 groups (*P*>0.05). By contrast, the levels of UA (40.46±8.93 μmol/L versus 49.88±18.45 μmol/L, *P*<0.05), NO (94.99±14.24 μmol/L versus 137.08±25.43 μmol/L, *P*<0.05), XO (13.40±2.43 U/L versus 48.11±17.01 U/L, *P*<0.05) and MDA (9.61±2.22 nmol/mL versus 14.24±2.69 nmol/mL, *P*<0.05) were elevated in the DM group, attenuated by allopurinol treatment (UA, 49.88±18.45 μmol/L versus 43.4±7.14 μmol/L; NO, 137.08±25.43 μmol/L versus 113.16±16.87 μmol/L; XO, 48.11±17.01 U/L versus 29.75±12.64 U/L and MDA nmol/mL, 14.24±2.69 nmol/mL versus 10.51±1.95 nmol/mL, *P*<0.05). Serum superoxide dismutase activity in the DM group was also higher but this did not reach statistical significance (471.30±66.89 U/mL versus 447.32±63.86 U/mL, *P*>0.05).

**Table 3:** Serum Biochemical and Oxidative Stress Parameters

	Control Group (n=10)	DM Group (n=10)	ALLO Group (n=10)
BUN, mmol/L	6.45±1.35	7.41±1.27	7.27±1.04
Cr, μmol/L	96.64±14.02	104.09±11.77	101.41±16.86
UA, μmol/L	40.46±8.93	49.88±18.45^a^	43.4±7.14^b^
TG, mmol/L	1.00±0.15	1.33±0.57	1.10±0.56
TC, mmol/L	1.69±0.18	1.81±0.46	1.76±0.56
HDL‐c, mmol/L	0.89±0.33	0.87±0.31	0.95±0.20
LDL‐c, mmol/L	0.56±0.12	0.69±0.31	0.66±0.37
INS, μIU/mL	15.88±2.44	7.27±1.63^a^	7.04±1.47
NO, μmol/L	94.99±14.24	137.08±25.43^a^	113.16±16.87^b^
SOD, U/mL	447.32±63.86	471.30±66.89	538.56±64.72^b^
XOD, U/L	13.40±2.43	48.11±17.01^a^	29.75±12.64^b^
MDA, nmol/mL	9.61±2.22	14.24±2.69^a^	10.51±1.95^b^

Values are mean±SD. BUN indicates blood urea nitrogen; Cr, creatinine; DM, diabetes mellitus; HDL‐c, high density lipoprotein cholesterol; INS, insulin; LDL‐c, low‐density lipoprotein cholesterol; MDA, malondialdehyde; NO, nitric oxide; SOD, superoxide dismutase; TC, cholesterol; TG, triglyceride; UA, uric acid; XOD, xanthine oxidase.

a Compared with the control group *P<*0.05.

b Compared with the DM group *P<*0.05.

**Figure 2:**
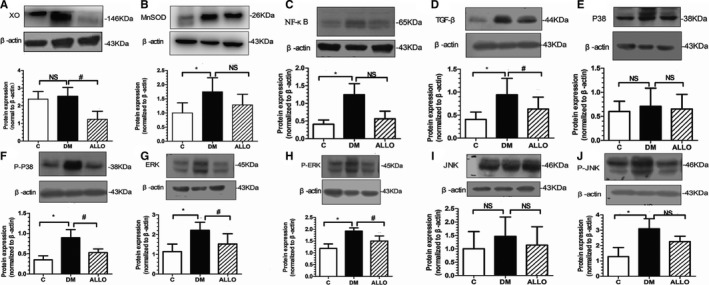
Oxidative stress and fibrosis related proteins expression in LA tissue. DM indicates diabetes mellitus; LA, left atrium. Protein levels were normalized to β‐actin. Xanthine oxidase (XO; A), MnSOD (manganese superoxide dismutase, B), NF‐κB (nuclear factor κ‐B, C), TGF‐β (transforming growth factor β, D), P38 (p38 mitogen‐activated protein kinases, E), P‐p38 (phosphorylated p38 mitogen‐activated protein kinases, F), ERK (extracellular signal‐regulated kinase, G), P‐ERK (phosphorylated extracellular signal‐regulated kinase, H), JNK (c‐Jun N‐terminal kinase, I), P‐JNK (phosphorylated c‐Jun Ntermialkinase, J). Data are presented as mean±SD. *Compared with the control group *P*<0.05. ^#^Compared with the DM group *P*<0.05. DM indicates diabetes mellitus; LA, left atrium; NS, not significant.

Western blot analyses were subsequently performed. These revealed increased oxidative stress markers including XO and manganese superoxide dismutase in the DM group compared with the control group, ameliorated by allopurinol treatment (Figures 2A and 2B). Similar patterns of changes were observed for atrial fibrosis‐related proteins, including NF‐κb, TGF‐ β, P‐p38, P‐JNK, ERK and P‐ERK. No significant differences in the expression of P38, JNK were found among the 3 groups (*P*>0.05).

### The Expression of Mitochondrial Related Protein in Diabetic Atrium

**Figure 3:**
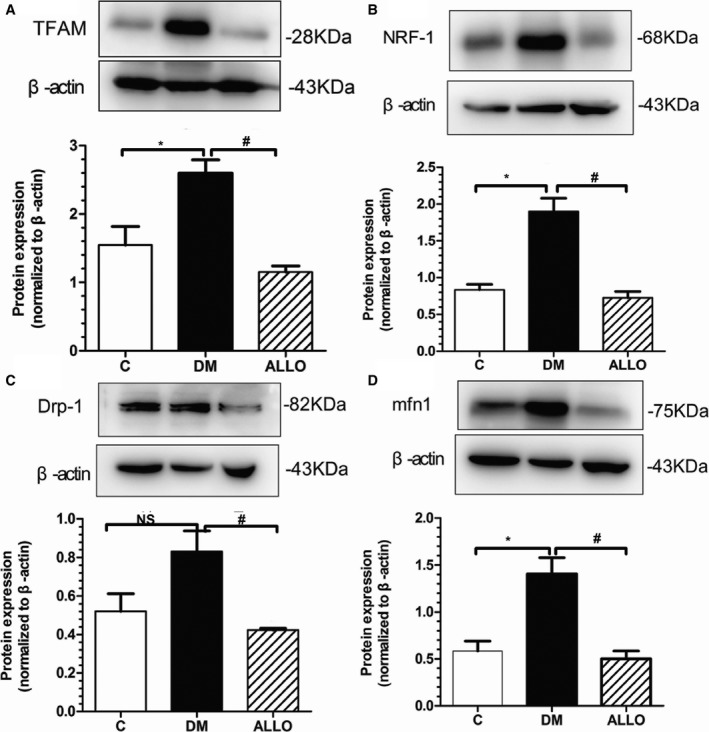
Mitochondrial‐related protein expression in LA tissue. A through D, Representative Western blot analysis of the protein expression in the 3 groups. (A) TFAM, (B) NRF‐1, (C) Drp‐1, (D) mfn1. Data are means±SD, relative protein level was normalized to β‐actin. *Compared with the control group *P*<0.05. ^#^Compared with the DM group *P*<0.05. DM indicates diabetes mellitus; NS, not significant; TFAM, mitochondrial transcription factor A.

Figure 3 showed the representative images of mitochondrial related protein. Besides Drp‐1, mitochondrial transcription factor A, nuclear respiratory factor‐1,and mfn1 were significantly increased in DM group (Figures 3A, 3B, and 3D), and interestingly all the proteins were downregulated distinctly by allopurinol application compared with the DM group, indicating the xanthine oxidase inhibitor may improve mitochondrial function.

### I_CaL_ Density, [Ca^2+^]_i_ Transients and Expression of Calcium Handling Proteins

**Figure 4:**
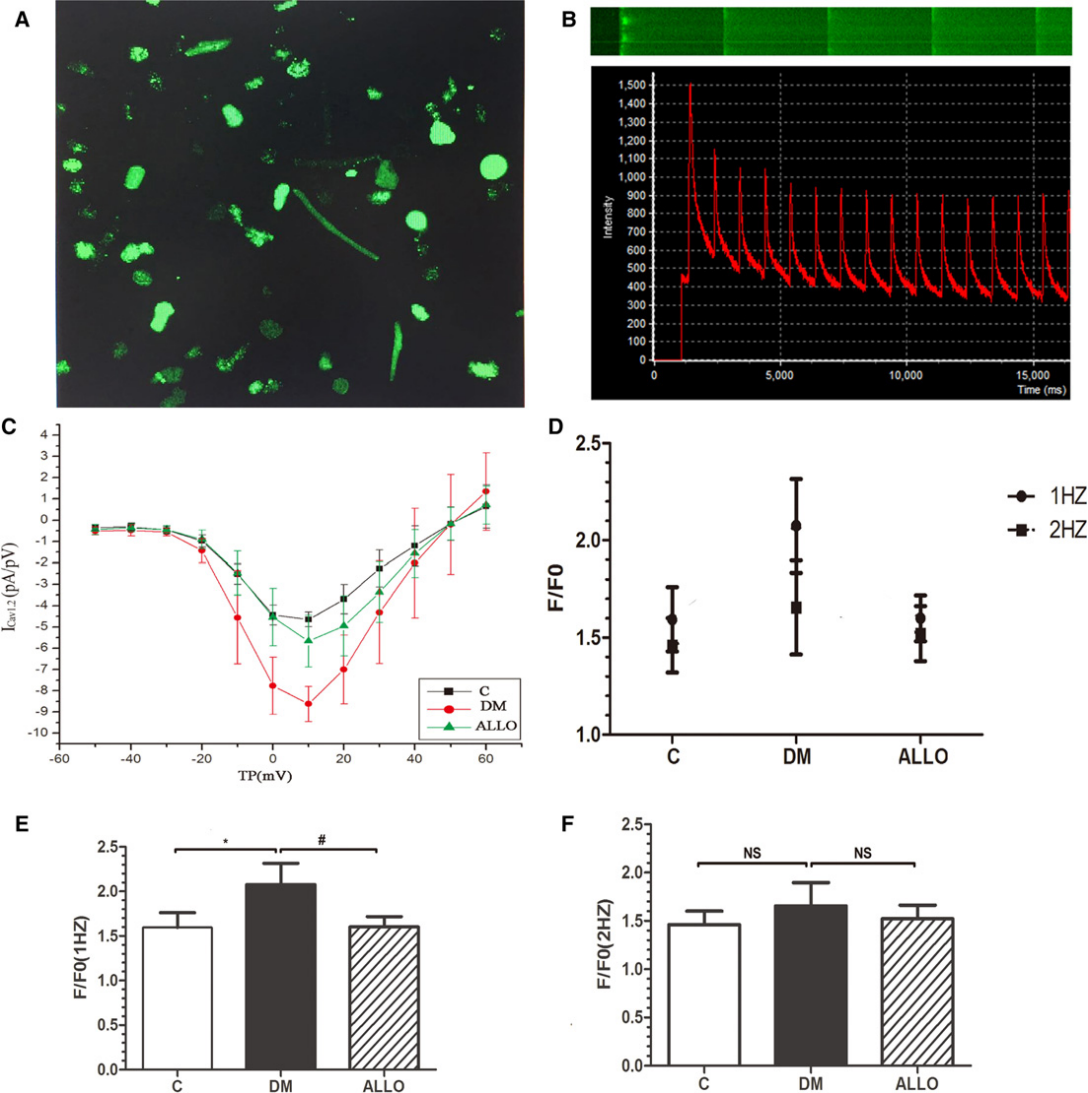
Current‐voltage (I–V) curve for ICaL density obtained using patch clamping (C). [Ca^2+^]_i_ transients measured by confocal microscopy (A, B, D through F). Representative images by flat scanning (A) and line scanning (B upper) and representative curve graph of [Ca^2+^]_i_ transients (B bottom). The mean magnitudes of [Ca^2+^]_i_ transients during 1 Hz (E) or 2 Hz (F) field stimulation and comparison of them (D). Data are presented as mean±SD. *Compared with the control group *P*<0.05. ^#^Compared with DM group *P*<0.05. DM indicates diabetes mellitus; NS, not significant.

The current‐voltage (I–V) curves for I_CaL_ density were obtained by applying a series of step depolarizing pulses from a holding potential of −50 to 60 mV in 10 mV increments for a 200 ms duration (Figure 4C). The maximum value of I_CaL_ density was observed when the voltage was set at 10 mV for all 3 groups. Compared with the control group (n=8), the DM group (n=8) did not show different morphology in the I–V curve but had larger I_CaL_ at all voltage steps tested between −30 to +65 mV (*P*<0.05), attenuated by allopurinol treatment (n=8; *P*<0.05).

Representative images of flat scanning and line scanning as well as curve graph of [Ca^2+^]_i_ transients were shown in Figures 4A and 4B. Peak [Ca^2+^]_i_ transients were assessed at different pacing frequencies. At 1 Hz, significantly higher transients were observed in the DM group compared with the control group (F/F_0_, 1.60±0.17 versus 2.08±0.24, *P*<0.05), and reduced by allopurinol (Figure 4E, 2.08±0.24 versus 1.60±0.12, *P*<0.05). By contrast, these differences were not observed when the pacing frequency was increased to 2 Hz (Figure 4F, 1.46±0.14, 1.66±0.24 and 1.52±0.14, respectively, *P*>0.05).

**Figure 5:**
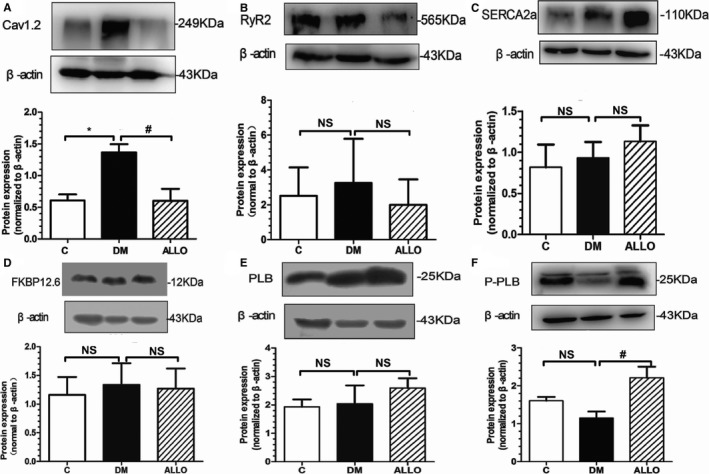
Expression of major Ca^2+^‐regulating proteins in left atrial tissue. Representative Western blot analysis of the protein expression in the 3 groups (A through F). Cav1.2 (A), RyR2 (B), SERCA2a (C), FKBP12.6 (D), PLB (E), P‐PLB (F). Data are presented as mean±SD. * and ^#^ indicate significant difference at *P*<0.05 when compared with the control group and the DM group, respectively. DM indicates diabetes mellitus; NS, not significant.

Expression of the major Ca^2+^‐regulating proteins involved in excitation–contraction coupling was probed by western blot of LA tissue homogenates. As shown in Figure 5A and 5B, the expression of RyR2 and Cav1.2 was increased in DM group and allopurinol ameliorated this alteration, although the change in RyR2 expression did not reach statistical significance, which was in accordance with the changes of I_CaL_ density and [Ca^2+^]_i_ transients. By contrast, no significant difference in FKBP12.6, the regulatory protein of RyR2, was observed (Figure 5D). The expression of both sarcoplasmic reticulum Ca^2+^‐ATPase SERCA2a and its regulatory protein PLB tended to be elevated in the ALLO group compared with the DM group without reaching statistical significance (Figures 5C and 5E). It is worth mentioning that the phosphorylation of PLB was significantly higher by allopurinol, which was closely related to the activity of SERCA2a (Figure 5F).

## Discussion

In this study, we examined the effects of the xanthine oxidase inhibitor allopurinol on electrical and structural remodeling in rabbit diabetes mellitus model. The major findings are as follow: diabetes mellitus induced (1) structural remodeling of the left ventricle; (2) a higher incidence of AF associated with prolonged IACT, AERPD and cycle length of atrial–ventricular conduction; (3) increased left atrial interstitial fibrosis; (4) significantly increased expression of NO, MDA, and XO levels; (5) higher protein expression levels of oxidative stress and atrial fibrosis, including XO, manganese superoxide dismutase, NF‐κB, TGF‐β, P‐p38, P‐JNK, ERK and P‐ERK (6) higher expression of mitochondrial‐related protein; (7) higher I_CaL_ density, [Ca^2+^]_I_ transients and L‐tpe calcium channel protein expression. Most of these abnormalities were attenuated by allopurinol treatment.

Several pathophysiological mechanisms underlying the association between AF and DM have been identified. These include autonomic modulation,^19^ electrical and structural remodeling, increased oxidative stress^20^, ^21^, ^22^, ^23^ and glycemic fluctuations.^24^, ^25^ Autonomic dysfunction in DM patients can be caused by hyperglycemia‐mediated pathways such as formation of advanced glycation end products (AGEs), elevated oxidative or nitrosative stress with increased production of free radicals.^19^ Our previous work has reported that in the diabetic atria, electrical remodeling took the form of characterized by conduction slowing, increased AERP dispersion and prolonged action potential duration, in an absence of frequency‐dependent shortening of action potential duration, and increased amplitude of action potential duration alternans.^21^, ^26^ These would be expected to predispose to re‐entrant arrhythmias. The common arrhythmogenic mechanism in diabetes mellitus is reentry, which can arise from abnormalities in conduction or repolarization or both. Repolarization abnormalities can result in early or delayed afterdepolarizations, which can initiate triggered activity when their magnitudes are sufficiently large to reach the threshold potential for sodium channel reactivation. They can also increase the dispersion of repolarization, promoting unidirectional conduction block and reentry.^27^, ^28^

Several oxidative stress mechanisms have been proposed to explain the development and perpetuation of AF in the context of diabetes mellitus.^29^ An excellent study demonstrated that maximal capacity for mitochondrial oxidation of palmitoyl‐carnitine is decreased while mitochondrial H_2_O_2_ emission during oxidation of carbohydrate‐ and lipid‐based substrates is increased, corresponding to increased local oxidative stress in the tissue, demonstrating mitochondria to be the main source of ROS.^23^ Mitochondrial transcription factor A and nuclear respiratory factor‐1 are key activators of mitochondrial transcription which also function as key genes involved in mitochondrial genome replication. Drp‐1 is a GTPase that regulates mitochondrial fission. Drp‐1 dysfunction can induce excessive fission, resulting in fragmented mitochondria more capable of producing ROS, thereby disrupting the intracellular regulatory processes.^30^ Mfn1 is a mitochondrial membrane protein acting as a mediator of mitochondrial fusion, interacting with mfn2 to facilitate mitochondrial targeting. In our study, all of the mitochondrial proteins mentioned above were increased in the diabetes mellitus group. This was possibly the consequence of stress response for the hypermetabolic state in diabetes mellitus, which were mitigated by the antioxidation of allopurinol, suggesting improvement in mitochondrial function.

The transcription factor nuclear factor‐kappa B (NF‐κB), via alterations in angiotensin and redox signaling, enhances heterogeneity in conduction, thereby promoting intra‐atrial reentry.^31^, ^32^ Diabetic hyperglycemia leads to overproduction of ROS, leading to NF‐κB upregulation, in turn promoting the transcription of proinflammatory genes.^33^, ^34^ Together, NF‐κB‐mediated vascular inflammation, oxidative stress, vascular and myocardial dysfunction in diabetes mellitus play a pivotal role in the genesis of AF. Using rabbits with diabetes mellitus, we found that serum oxidative stress parameters including NO, MDA, and XO, as well as protein expression of XO and NF‐κB were significantly increased in the DM group.

Ang II mediates atrial fibrosis by provoking mitogen activated protein kinases (MAPKs), promoting endogenous synthesis of TGF‐β1 and connective tissue growth factor (CTGF).^35^, ^36^, ^37^, ^38^, ^39^, ^40^ MAPKs are pivotal mediators of the effects of Ang II on tissue structure by increasing TGF‐β1 expression, leading to atrial fibrosis.^38^, ^39^, ^40^ TGF‐β1 is a cytokine that regulates cell proliferation, apoptosis, migration, and synthesis of ECM, such as fibronectin and collagen in the atrium.^41^, ^42^ In our DM rabbit model, we found that hyperglycemia enhanced the activation of MAPKs (p38MAPK, ERK, JNK), expression of TGF‐β1 as well as atrial fibrosis, in keeping with findings from our previous study.^26^

Calcium homeostasis is an important determinant of action potential duration, excitation–contraction coupling and the generation of contractile activity. Early and delayed afterdepolarizations, which are secondary depolarization events occurring before the subsequent AP, can initiate arrhythmias. Early after depolarizations are typically generated when the repolarization phase of the cardiac AP is prolonged, leading to reactivation of the L‐type Ca^2+^ channels (I_Ca_) or activation of the Na^+^‐Ca^2+^ exchanger (I_NCX_) secondary to spontaneous Ca^2+^ release from the sarcoplasmic reticulum. Delayed afterdepolarizations are associated with Ca^2+^ overload, which activates several Ca^2+^‐sensitive currents, which together constitute the transient in ward current (I_TI_).^43^ In addition, Ca^2+^ alternans can be induced in cardiac cells by making sarcoplasmic release of calcium strongly dependent upon the SR Ca^2+^ content. Compelling evidence suggests that action potential duration alternans, whether in the atria or the ventricles, are driven by Ca^2+^ alternans, which are attributable to ryanodine receptor refractoriness.^44^ Moreover, calcium homeostasis is an important regulator of mitochondrial synthesis. Mitochondria are often located adjacent to the junctional SR, and it has therefore been suggested that Ca^2+^ release will increase the local Ca^2+^ concentration to a high level within the mitochondria. This acts as an important activator signal for different mitochondrial enzymes leading to increased ATP generation.^45^ We found higher I_CaL_ and Ca^2+^ transients were increased in DM group with an enhanced expression of Ca^2+^ handling proteins including Cav1.2 and RyR2, leading to a rise of [Ca^2+^]_i_ from the extracellular space and the SR. Furthermore, the fact that SERCA2a was slightly decreased in DM group with the reduce of P‐PLB expression, which may attenuate its activity, would impair Ca^2+^ uptake of SR and lead to intracellular Ca^2+^ overload.

The association between hyperuricemia and AF is well‐established. Accumulation of uric acid inside atrial cardiomyocytes can induce remodeling by elevating ROS levels. Several previous studies have confirmed that reduction of oxidative stress through inhibition of xanthine oxidase using allopurinol or other similar drug, of NADPH‐oxidase using apocynin,^21^ or the use of N‐acetylcysteine^46^ is beneficial to atrial remodeling. Xanthine oxidoreductase (XOR), a key enzyme of purine catabolism, consists of two interconvertible forms, xanthine dehydrogenase (XDH) and xanthine oxidase (XO). It is responsible for the generation of free radicals in the terminal steps in purine metabolism, which have previously been associated with higher vulnerability to developing AF.^14^ Allopurinol, a selective XO inhibitor, has been demonstrated to reverse structural remodeling in the failing ventricle.^47^, ^48^ Allopurinol prevented hyperglycemia‐induced oxidative stress, cardiomyocyte hypertrophy and cardiac fibrosis and attenuated cardiac dysfunction,^15^ in which prevention of ROS‐induced oxidative stress and reduction of myocardial collagen formation may represent one of the major pathogenic mechanisms. Zhang et al found that DPP‐4 inhibitors decreased mitochondrial ROS production rate and ameliorate arrhythmic substrate in a DM rabbit model.^49^ In another study, Zou et al illustrated that ranolazine inhibits oxidative stress and improves mitochondrial function in the atrium of acetylcholine‐CaCl_2_–induced atrial fibrillation rats partly by increasing the expression of antioxidants such as GSH‐Px and manganese superoxide dismutase to reduce ROS levels in mitochondria.^50^ Furthermore, in a canine model subjected to atrial tachycardia pacing (ATP, 400 bpm) without atrioventricular block for 4 weeks leading to left ventricular dysfunction, Sakabe et al evaluated the dynamics of atrial‐tachycardia remodeling. In placebo‐treated control dogs, 4 weeks of ATP significantly increased AF duration and decreased atrial effective refractory period, which were attenuated by allopurinol through preventing atrial electrical and structural remodeling. This is the first report to show the preventive effects of allopurinol on AF.^14^ Our results complement these previous findings by demonstrating that allopurinol markedly inhibited atrial structural remodeling, increased vulnerability to AF and the expression of NF‐κB, TGF‐β, P‐p38, P‐JNK, ERK and P‐ERK, serum oxidative stress parameters (NO, XOD and MDA) as well as the abnormity of calcium homeostasis. These findings suggest that the inhibition of ROS formation is a critical step in preventing AF in rabbits with diabetes mellitus.

### Study Limitations

Some limitations of this study should be noted. As the main aim of this study was to examine the relationship between oxidative stress, structural remodeling, and calcium homeostasis; activities of non‐calcium–related ion channels were not examined. Further studies are required to elucidate their roles in AF development of our model. Moreover, the potential beneficial effects of allopurinol for preventing AF development in humans should be examined in future studies.

## Conclusions

Our study demonstrates that allopurinol attenuated atrial structural and electrical remodeling and suppresses AF vulnerability in alloxan‐induced rabbits with diabetes mellitus. These protective effects were associated with reductions in ROS formation and atrial fibrosis‐related factors and abnormal calcium homeostasis. These findings suggest that allopurinol may be useful for treating diabetes mellitus‐related AF.

## Sources of Funding

This work was supported by grants (81570298, 81270245, 30900618 to Liu) from the National Natural Science Foundation of China, Tianjin Natural Science Foundation (16JCZDJC34900 to Liu). Tse received support through a clinical assistant professorship from the Croucher Foundation of Hong Kong.

## Disclosures

None.

## Supporting information


**Table S1.** Primary Antibodies of Proteins

## References

[1] Benjamin EJ , Wolf PA , D'Agostino RB , Silbershatz H , Kannel WB , Levy D . Impact of atrial fibrillation on the risk of death: the Framingham Heart Study. Circulation. 1998;98:946–952. 9737513 10.1161/01.cir.98.10.946

[2] Van den Berg MP , van Gelder IC , van Veldhuisen DJ . Impact of atrial fibrillation on mortality in patients with chronic heart failure. Eur J Heart Fail. 2002;4:571–575. 12413498 10.1016/s1388-9842(02)00094-6

[3] Goudis CA , Korantzopoulos P , Ntalas IV , Kallergis EM , Liu T , Ketikoglou DG . Diabetes mellitus and atrial fibrillation: pathophysiological mechanisms and potential upstream therapies. Int J Cardiol. 2015;184:617–622. 25770841 10.1016/j.ijcard.2015.03.052

[4] Ziolo MT , Mohler PJ . Defining the role of oxidative stress in atrial fibrillation and diabetes. J Cardiovasc Electrophysiol. 2015;26:223–225. 25298131 10.1111/jce.12560 PMC4323889

[5] Zhang Q , Liu T , Ng CY , Li G . Diabetes mellitus and atrial remodeling: mechanisms and potential upstream therapies. Cardiovasc Ther. 2014;32:233–241. 25065462 10.1111/1755-5922.12089

[6] Tadic M , Cuspidi C . Type 2 diabetes mellitus and atrial fibrillation: from mechanisms to clinical practice. Arch Cardiovasc Dis. 2015;108:269–276. 25858534 10.1016/j.acvd.2015.01.009

[7] Berry CE , Hare JM . Xanthine oxidoreductase and cardiovascular disease: molecular mechanisms and pathophysiological implications. J Physiol. 2004;555:589–606. 14694147 10.1113/jphysiol.2003.055913 PMC1664875

[8] Isabelle M , Vergeade A , Moritz F , Dautraux B , Henry JP , Lallemand F , Richard V , Mulder P , Thuillez C , Monteil C . NADPH oxidase inhibition prevents cocaine‐induced up‐regulation of xanthine oxidoreductase and cardiac dysfunction. J Mol Cell Cardiol. 2007;42:326–332. 17217956 10.1016/j.yjmcc.2006.11.011

[9] Bers DM . Calcium cycling and signaling in cardiac myocytes. Annu Rev Physiol. 2008;70:23–49. 17988210 10.1146/annurev.physiol.70.113006.100455

[10] Eisner DA , Kashimura T , Venetucci LA , Trafford AW . From the ryanodine receptor to cardiac arrhythmias. Circ J. 2009;73:1561–1567. 19667488 10.1253/circj.cj-09-0478

[11] Nattel S , Dobrev D . The multidimensional role of calcium in atrial fibrillation pathophysiology: mechanistic insights and therapeutic opportunities. Eur Heart J. 2012;33:1870–1877. 22507975 10.1093/eurheartj/ehs079

[12] Eisner D , Bode E , Venetucci L , Trafford A . Calcium flux balance in the heart. J Mol Cell Cardiol. 2013;58:110–117. 23220128 10.1016/j.yjmcc.2012.11.017

[13] Minhas KM , Saraiva RM , Schuleri KH , Lehrke S , Zheng M , Saliaris AP , Berry CE , Barouch LA , Vandegaer KM , Li D , Hare JM . Xanthine oxidoreductase inhibition causes reverse remodeling in rats with dilated cardiomyopathy. Circ Res. 2006;98:271–279. 16357304 10.1161/01.RES.0000200181.59551.71

[14] Sakabe M , Fujiki A , Sakamoto T , Nakatani Y , Mizumaki K , Inoue H . Xanthine oxidase inhibition prevents atrial fibrillation in a canine model of atrial pacing‐induced left ventricular dysfunction. J Cardiovasc Electrophysiol. 2012;23:1130–1135. 22587612 10.1111/j.1540-8167.2012.02356.x

[15] Gao X , Xu Y , Xu B , Liu Y , Cai J , Liu HM , Lei S , Zhong YQ , Irwin MG , Xia Z . Allopurinol attenuates left ventricular dysfunction in rats with early stages of streptozotocin‐induced diabetes. Diabetes Metab Res Rev. 2012;28:409–417. 22389139 10.1002/dmrr.2295

[16] Saavedra WF , Paolocci N , St John ME , Skaf MW , Stewart GC , Xie JS , Harrison RW , Zeichner J , Mudrick D , Marbán E , Kass DA , Hare JM . Imbalance between xanthine oxidase and nitric oxide synthase signaling pathways underlies mechanoenergetic uncoupling in the failing heart. Circ Res. 2002;90:297–304. 11861418 10.1161/hh0302.104531

[17] Farquharson CA , Butler R , Hill A , Belch JJ , Struthers AD . Allopurinol improves endothelial dysfunction in chronic heart failure. Circulation. 2002;106:221–226. 12105162 10.1161/01.cir.0000022140.61460.1d

[18] Li D , Melnyk P , Feng J , Wang Z , Petrecca K , Shrier A , Nattel S . Effects of experimental heart failure on atrial cellular and ionic electrophysiology. Circulation. 2000;101:2631–2638. 10840016 10.1161/01.cir.101.22.2631

[19] Pop‐Busui R . Cardiac autonomic neuropathy in diabetes: a clinical perspective. Diabetes Care. 2010;33:434–441. 20103559 10.2337/dc09-1294 PMC2809298

[20] Liu C , Fu H , Li J , Yang W , Cheng L , Liu T , Li G . Hyperglycemia aggravates atrial interstitial fibrosis, ionic remodeling and vulnerability to atrial fibrillation in diabetic rabbits. Anadolu Kardiyol Derg. 2012;12:543–550. 22877897 10.5152/akd.2012.188

[21] Qiu J , Zhao J , Li J , Liang X , Yang Y , Zhang Z , Zhang X , Fu H , Korantzopoulos P , Liu T , Li G . NADPH oxidase inhibitor apocynin prevents atrial remodeling in alloxan‐induced diabetic rabbits. Int J Cardiol. 2016;221:812–819. 27434350 10.1016/j.ijcard.2016.07.132

[22] Kato T , Yamashita T , Sekiguchi A , Sagara K , Takamura M , Takata S , Kaneko S , Aizawa T , Fu LT . What are arrhythmogenic substrates in diabetic rat atria? J Cardiovasc Electrophysiol. 2006;17:890–894. 16759295 10.1111/j.1540-8167.2006.00528.x

[23] Anderson EJ , Kypson AP , Rodriguez E , Anderson CA , Lehr EJ , Neufer PD . Substrate‐specific derangements in mitochondrial metabolism and redox balance in the atrium of the type 2 diabetic human heart. J Am Coll Cardiol. 2009;54:1891–1898. 19892241 10.1016/j.jacc.2009.07.031 PMC2800130

[24] Lip G , Varughese G . Diabetes mellitus and atrial fibrillation: perspectives on epidemiological and pathophysiological links. Int J Cardiol. 2005;105:319–321. 16274776 10.1016/j.ijcard.2005.03.003

[25] Saito S , Teshima Y , Fukui A , Kondo H , Nishio S , Nakagawa M , Saikawa T , Takahashi N . Glucose fluctuations increase the incidence of atrial fibrillation in diabetic rats. Cardiovasc Res. 2014;104:5–14. 25082849 10.1093/cvr/cvu176

[26] Fu H , Li G , Liu C , Li J , Wang X , Cheng L , Liu T . Probucol prevents atrial remodeling by inhibiting oxidative stress and TNF‐α/NF‐κB/TGF‐βsignal transduction pathway in alloxan‐induced diabetic rabbit. J Cardiovasc Electrophysiol. 2015;26:211–222. 25199622 10.1111/jce.12540

[27] Choy L , Yeo JM , Tse V , Chan SP , Tse G . Cardiac disease and arrhythmogenesis: mechanistic insights from mouse studies. Int J Cardiol Heart Vasc. 2016;12:1–10. 27766308 10.1016/j.ijcha.2016.05.005 PMC5064289

[28] Tse G , Lai ET , Tse V , Yeo JM . Molecular and electrophysiological mechanisms underlying cardiac arrhythmogenesis in diabetes mellitus. J Diabetes Res. 2016;2016:2848759. 27642609 10.1155/2016/2848759 PMC5011530

[29] Rochette L , Zeller M , Cottin Y , Vergely C . Diabetes, oxidative stress and therapeutic strategies. Biochim Biophys Acta. 2014;1840:2709–2729. 24905298 10.1016/j.bbagen.2014.05.017

[30] Sharp WW , Archer SL . Mitochondrial dynamics in cardiovascular disease: fission and fusion foretell form and function. J Mol Med (Berl). 2015;93:225–228. 25669447 10.1007/s00109-015-1258-2 PMC4338002

[31] Kabe Y , Ando K , Hirao S , Yoshida M , Handa H . Redox regulation of NF‐kappa B activation: distinct redox regulation between the cytoplasm and the nucleus. Antioxid Redox Signal. 2005;7:395–403. 15706086 10.1089/ars.2005.7.395

[32] Lorenzo O , Picatoste B , Ares‐Carrasco S , Ramírez E , Egido J , Tuñón J . Potential role of nuclear factor kappaB in diabetic cardiomyopathy. Mediators Inflamm. 2011;2011:652097. 21772665 10.1155/2011/652097 PMC3136091

[33] Hall G , Hasday JD , Rogers TB . Regulating the regulator: NF‐kappaB signaling in heart. J Mol Cell Cardiol. 2006;41:580–591. 16949095 10.1016/j.yjmcc.2006.07.006

[34] Freund C , Schmidt‐Ullrich R , Baurand A , Dunger S , Schneider W , Loser P , El‐Jamali A , Dietz R , Scheidereit C , Bergmann MW . Requirement of nuclear factor‐kappa B in angiotensin II‐ and isoproterenol induced cardiac hypertrophy in vivo. Circulation. 2005;111:2319–2325. 15870116 10.1161/01.CIR.0000164237.58200.5A

[35] Kupfahl C , Pink D , Friedrich K , Zurbrügg HR , Neuss M , Warnecke C , Fielitz J , Graf K , Fleck E , Regitz‐Zagrosek V . Angiotensin II directly increases transforming growth factor beta1and osteopontin and indirectly affects collagen mRNA expression in the human heart. Cardiovasc Res. 2000;46:463–475. 10912457 10.1016/s0008-6363(00)00037-7

[36] Ko WC , Hong CY , Hou SM , Lin CH , Ong ET , Lee CF , Tsai CT , Lai LP . Elevated expression of connective tissue growth factor in human atrial fibrillation and angiotensin II‐treated cardiomyocytes. Circ J. 2011;75:1592–1600. 21576830 10.1253/circj.cj-10-0892

[37] Tsai CT , Tseng CD , Hwang JJ , Wu CK , Yu CC , Wang YC , Chen WP , Lai LP , Chiang FT , Lin JL . Tachycardia of atrial myocytes induces collagen expression in atrial fibroblasts through transforming growth factor β1. Cardiovasc Res. 2011;89:805–815. 21134900 10.1093/cvr/cvq322

[38] Li L , Fan D , Wang C , Wang JY , Cui XB , Wu D , Zhou Y , Wu LL . Angiotensin II increases periostin expression via Ras/p38 MAPK/CREB and ERK1/2/TGF‐β1 pathways in cardiac fibroblasts. Cardiovasc Res. 2011;91:80–89. 21367774 10.1093/cvr/cvr067

[39] Naito T , Masaki T , Nikolic‐Paterson DJ , Tanji C , Yorioka N , Kohno N . Angiotensin II induces thrombospondin‐1 production in human mesangial cells via p38 MAPK and JNK: a mechanism for activation of latent TGF‐beta1. Am J Physiol Renal Physiol. 2004;286:F278–F287. 14583433 10.1152/ajprenal.00139.2003

[40] Liu B , Yu J , Taylor L , Zhou X , Polgar P . Microarray and phosphokinase screenings leading to studies on ERK and JNK regulation of connective tissue growth factor expression by angiotensin II 1a and bradykinin B2 receptors in Rat1 fibroblasts. J Cell Biochem. 2006;97:1104–1120. 16294326 10.1002/jcb.20709

[41] Lijnen PJ , Petrov VV , Fagard RH . Induction of cardiac fibrosis by transforming growth factor‐beta(1). Mol Genet Metab. 2000;71:418–435. 11001836 10.1006/mgme.2000.3032

[42] Khan R , Sheppard R . Fibrosis in heart disease: understanding the role of transforming growth factor‐beta in cardiomyopathy, valvular disease and arrhythmia. Immunology. 2006;118:10–24. 16630019 10.1111/j.1365-2567.2006.02336.x PMC1782267

[43] Tse G , Lai ET , Lee AP , Yan BP , Wong SH . Electrophysiological mechanisms of gastrointestinal arrhythmogenesis: lessons from the heart. Front Physiol. 2016;7:230. 27378939 10.3389/fphys.2016.00230 PMC4906021

[44] Tse G , Wong ST , Tse V , Lee YT , Lin HY , Yeo JM . Cardiac dynamics: alternans and arrhythmogenesis. J Arrhythm. 2016;32:411–417. 27761166 10.1016/j.joa.2016.02.009 PMC5063258

[45] Lu X , Ginsburg KS , Kettlewell S , Bossuyt J , Smith GL , Bers DM . Measuring local gradients of intramitochondrial [Ca(2+)] in cardiac myocytes during sarcoplasmic reticulum Ca(2+) release. Circ Res. 2013;112:424–431. 23243207 10.1161/CIRCRESAHA.111.300501 PMC3566246

[46] Orenes‐Piñero E , Valdés M , Lip GY , Marín F . A comprehensive insight of novel antioxidant therapies for atrial fibrillation management. Drug Metab Rev. 2015;47:388–400. 27412960 10.3109/03602532.2015.1077858

[47] Cappola TP , Kass DA , Nelson GS , Berger RD , Rosas GO , Kobeissi ZA , Marbán E , Hare JM . Allopurinol improves myocardial efficiency in patients with idiopathic dilated cardiomyopathy. Circulation. 2001;104:2407–2411. 11705816 10.1161/hc4501.098928

[48] Amado LC , Saliaris AP , Raju SV , Lehrke S , St John M , Xie J , Stewart G , Fitton T , Minhas KM , Brawn J , Hare JM . Xanthine oxidase inhibition ameliorates cardiovascular dysfunction in dogs with pacing‐induced heart failure. J Mol Cell Cardiol. 2005;39:531–536. 15963530 10.1016/j.yjmcc.2005.04.008

[49] Zhang X , Zhang Z , Zhao Y , Jiang N , Qiu J , Yang Y , Li J , Liang X , Wang X , Tse G , Li G , Liu T . Alogliptin, a dipeptidyl peptidase‐4 inhibitor, alleviates atrial remodeling and improves mitochondrial function and biogenesis in diabetic rabbits. J Am Heart Assoc. 2017;6:e005945 DOI: 10.1161/JAHA.117.005945. 28507060 10.1161/JAHA.117.005945 PMC5524117

[50] Zou D , Geng N , Chen Y , Ren L , Liu X , Wan J , Guo S , Wang S . Ranolazine improves oxidative stress and mitochondrial function in the atrium of acetylcholine‐CaCl_2_ induced atrial fibrillation rats. Life Sci. 2016;156:7–14. 27208652 10.1016/j.lfs.2016.05.026

