# Supplementary material for: Xanthine Oxidase Inhibitor Allopurinol Prevents Oxidative Stress‐Mediated Atrial Remodeling in Alloxan‐Induced Diabetes Mellitus Rabbits
Source: J Am Heart Assoc. 2018 May 2;7(10):e008807. doi: 10.1161/JAHA.118.008807 (PMC6015332; doi:10.1161/JAHA.118.008807)
Supplement: Supplementary file 1 — Table S1. Primary Antibodies of Proteins [file JAH3-7-e008807-s001.pdf]

SUPPLEMENTAL MATERIAL

Table S1. Primary antibodies of proteins.

Proteins	Companies of primary antibodies	Item No
NF-κB	Abcam, USA	ab90523
TGF-β	Abcam, USA	ab190503
P38	Abcam, USA	ab131186
P-p38	Abcam, USA	ab4822
ERK	Abcam, USA	Unavailable*
P-ERK	Abcam,,USA	Unavailable*
JNK	Abcam, USA	ab10664
P-JNK	Abcam, USA	ab4821
XO	Abcam, USA	ab109235
MnSOD	Abcam, USA	ab13533
TFAM	Novus Biologicals	NBP2-19437
NRF-1	Abcam, USA	ab111746
Drp-1	Abcam, USA	ab140494
Mfn1	Abcam, USA	ab104274
Cav1.2	Abcam, USA	ab58552
RyR2	LifeSpan Biosciences	LS-C93425/61935
SERCA2a	Gene Tex	GTX22817
FKBP12.6	Abcam, USA	ab82316
PLB	Abcam, USA	ab2865
P-PLB	Abcam, USA	Ab15000

*The antibodies were given as a gift by others and the special details were unavailable.
